# Adopting common data elements (CDEs) for the National Trauma Research Repository (NTRR): the results of an outcome, outcome measures, and rehabilitation Delphi Survey

**DOI:** 10.1136/tsaco-2025-002088

**Published:** 2026-07-02

**Authors:** Kevin M Schuster, Ashley N Moreno, Dagmar Amtmann, Jill Marie Cancio, John Chovanes, Adele Doperalski, Eric Elster, Joseph T Giacino, Kirby Robert Gross, Juan Pablo Herrera-Escobar, Karen J Kowalske, Ingrid Parry, Jeffrey C Schneider, Olga Volk, The NTRR investigator group, Suresh Agarwal

**Affiliations:** 1 Surgery, Yale University School of Medicine, New Haven, Connecticut, USA; 2 Surgery, Bridgeport Hospital, Bridgeport, Connecticut, USA; 3 Coalition for National Trauma Research, San Antonio, Texas, USA; 4 Rehabilitation Medicine, University of Washington, Seattle, Washington, USA; 5 US Army Institute of Surgical Research Burn Center, Joint Base San Antonio Fort Sam Houston, Texas, USA; 6 Cooper University Health Care, Camden, New Jersey, USA; 7 National Institute of Neurological Disorders and Stroke, Bethesda, Maryland, USA; 8 Surgery, Uniformed Services University, Bethesda, Maryland, USA; 9 Physical Medicine and Rehabilitation, Spaulding Rehabilitation Hospital, Charlestown, Massachusetts, USA; 10 Physical Medicine and Rehabilitation, Harvard Medical School, Boston, Massachusetts, USA; 11 Surgery, Cooper University Hospital, Camden, New Jersey, USA; 12 Center for Surgery and Public Health, Brigham and Women’s Hospital, Boston, Massachusetts, USA; 13 Physical Medicine and Rehabilitation, The University of Texas Southwestern Medical Center, Dallas, Texas, USA; 14 Surgery, University of California Davis, Sacramento, California, USA; 15 Clinical Research, Shriners Hospitals, Northern California, Sacramento, California, USA; 16 BRICS Data Dictionary Curator, Publicis Sapient, Arlington, Virginia, USA

**Keywords:** outcomes, rehabilitation

## Abstract

**Introduction:**

The National Trauma Research Repository (NTRR) serves as the central warehouse for trauma clinical research data that result from military and civilian trauma research. An effort to enhance the NTRR was undertaken through the identification and integration of common data elements (CDEs). CDEs will improve usability for primary investigators and allow for enhanced secondary analysis and combing of NTRR archived studies. A consensus-driven approach was used to review established data elements and recommend trauma basic CDEs for the outcomes and rehabilitation environments of care for inclusion in the NTRR data dictionary.

**Methods:**

A multidisciplinary workgroup located and reviewed data dictionaries, codebooks, data collection forms, and published articles for outcome measurement instruments and individual outcome and rehabilitation data elements. Three rounds of a Delphi Survey were completed, and monthly meetings with the workgroup were conducted. Consensus during the Delphi Survey was identified with an 80% agreement threshold.

**Results:**

Fifteen sources were reviewed for outcome measurement instruments, 39 measurement instruments were presented for consideration in the Delphi Survey, and 12 instruments (28%) reached consensus for inclusion in the NTRR data dictionary. Seventeen sources were reviewed for individual outcome data elements, 55 data elements were included in the Delphi Survey, and 45 data elements reached consensus for inclusion. Eleven sources were reviewed for rehabilitation data elements. Of the 41 rehabilitation data elements identified and included in the Delphi, 17 data elements reached consensus for inclusion.

**Discussion:**

This workgroup selected outcome measurement instruments, outcome CDEs, and rehabilitation CDEs for inclusion in the NTRR data dictionary as basic CDEs. Next steps include disseminating these elements, integrating their use in trauma studies, and tracking the use and usability of these instruments and CDEs.

**Level of evidence:**

VII.

**WHAT IS ALREADY KNOWN ON THIS TOPIC**

- There is limited ability to perform secondary analysis and combine studies in the National Trauma Research Repository (NTRR) due to lack of standardized variable definitions and permissible values.

**WHAT THIS STUDY ADDS**

- This is a consensus-driven approach to creating standardized data elements and values in the NTRR.

**HOW THIS STUDY MIGHT AFFECT RESEARCH, PRACTICE OR POLICY**

- This work provides common data elements for secondary and combined analyses of NTRR studies.

## Introduction

 More than four million deaths were attributed to unintentional and violence-related injuries across the world in 2019, and it is estimated that these injuries account for 8% of years lived with disability.^1^ Trauma research remains hindered by a lack of standardized data elements that enable meaningful comparison and aggregation of findings across studies and institutions.^2^ The generalizability and reproducibility of trauma research, particularly within the field of outcomes and rehabilitation, is limited by the variability in how data are defined, collected, and reported.

Common data elements (CDEs) are standardized, precisely defined variables intended to be consistently collected and used across studies within a given field. The benefits of CDE use and data sharing, especially within trauma research, are numerous.^3 4^ Individual researchers benefit from data sharing via increased visibility and enhanced efficiency. The research community benefits from advances in reproducibility, improved long-term data archiving, and a reduction of unnecessary studies. Societal benefits from data sharing include enhanced innovation, easier access to research, and scientifically informed policy making. The ultimate goal of responsible sharing of clinical trial data is to increase scientific knowledge that leads to better therapies for patients.

The Delphi method is a systematic approach for achieving consensus among a panel of experts and is well suited for selecting CDEs. The iterative flow of the Delphi process allows for participant input, controlled feedback, and statistical aggregation of group responses, which helps to mitigate the influence of dominant individuals and supports balanced decision-making.

The National Trauma Research Repository (NTRR) was established to serve as the central warehouse for trauma clinical research data that results from military and civilian trauma research, whether federally or privately funded. As part of the development of this key piece of the national trauma research infrastructure, the NTRR was tasked with addressing the existing variability of data collection within trauma research and convened a series of expert workgroups to identify “basic” CDEs in specific study environments or phases of care. These basic CDEs are recommended to be collected in trauma studies being conducted within these specific therapeutic areas.

Outcomes and rehabilitation after traumatic injury were identified as specific care environments of interest for the NTRR, as mortality metrics alone do not capture the full extent of outcomes after traumatic injury. The need to collect and investigate both short-term and long-term outcomes after traumatic injury has been highlighted. Metrics to assess long-term functional outcomes have been developed through pivotal projects such as the Lower Extremity Assessment Project, the Functional Outcomes and Recovery after Trauma Emergencies project, and the Consensus Conference on Trauma Patient-Reported Outcome Measures (hosted January 10–11, 2019).^5^,^7^ The Major Extremity Trauma Research Consortium and the Coalition for National Trauma Research (CNTR) National Trauma Research Action Plan also prioritize metrics to assess long-term functional outcomes through standardized data points.^8^,^11^ Through these projects, investigators have amplified the importance of collecting data regarding survivors’ physical function and functional abilities, mental health status, participation in social life, as well as overall quality of life.

Rehabilitation of an injured patient occurs in various settings, including the acute and postacute care settings, and takes many forms, such as physical medicine and rehabilitation (PM&R) care, occupational therapy, physical therapy, and speech-language pathology. Research endeavors into the rehabilitation phase of care include the Spinal Cord Injury Rehabilitation Study, Traumatic Brain Injury (TBI) Practice-Based Evidence Study, and three model system programs sponsored by the National Institute on Disability, Independent Living, and Rehabilitation Research: the Burn Injury Model System, Traumatic Brain Injury Model System, and the Spinal Cord Injury Model System.^12^,^16^ Through these efforts, the frequency, intensity, activities, interventions, and specific components of rehabilitation programs have all been identified as important data elements to measure and track.

This article describes the systematic approach the NTRR Outcomes and Rehabilitation Workgroup used to identify and establish basic CDEs for inclusion in the NTRR data dictionary. Using a structured Delphi consensus process, the workgroup engaged a diverse panel of subject matter experts for trauma outcomes and rehabilitation to review, refine, and prioritize candidate CDEs. The goal of this process was to develop a consensus-driven set of data elements that would enhance the quality, consistency, and utility of outcomes and rehabilitation trauma research conducted using the NTRR. Together, basic CDEs for the outcomes and rehabilitation care environments begin to capture the experiences and recovery trajectory of major trauma survivors posthospitalization.

## Methods

Members of the Executive Steering and CDE Steering Committees recommended subject matter experts for the NTRR Outcomes and Rehabilitation Workgroup. The selection and membership of these two steering committees are described in previous articles.^17^ The Coalition for National Trauma Research (CNTR) gathered information on all those recommended to serve, including work environment, trauma subspecialty, research experience, geographic location, and military experience to comprise this workgroup with diverse perspectives from across the USA. After guidance from the National Institute of Health (NIH) regarding effective Delphi groups, we sought roughly a dozen members. In total, CNTR sent recruiting emails to 12 individuals for the Outcomes and Rehabilitation Workgroup; all 12 individuals accepted and participated in the consensus process. The workgroup was multidisciplinary and included experts in civilian and military trauma research, PM&R, long-term functional outcomes research, and burn research. Workgroup members represented university-based trauma centers, civilian and military rehabilitation centers, and the National Institute of Neurological Disorders and Stroke (NINDS). To identify the most commonly collected data elements from the two therapeutic areas, workgroup members collectively compiled relevant and applicable studies for review by the CNTR team. The team then located and reviewed data dictionaries, codebooks, data collection forms, and/or published articles related to the recommended sources.

The project team identified outcome measurement instruments and individual data elements from the sources and conducted a frequency analysis to determine the most commonly used measurement instruments and data elements collected for outcomes and rehabilitation. They selected data elements that would be applicable to the general adult population. The process for researching existing definitions and permissible values (PVs) for each of the data elements has been described previously.^17^ For the measurement instruments, workgroup members were provided with an overview of each instrument, including the cost, any licensing requirements, the clinical outcome assessment type, the number of items included in the instrument, the average completion time, and the instrument’s full citation.

The Delphi approach was selected for this project due to its iterative nature, the incorporation of feedback after each round, and the ability for participants to complete the process remotely and asynchronously. The ACcurate COnsensus Reporting Document checklist was followed in reporting this consensus-generating process (online supplemental item 1).^18^ Having gained extensive prior experience in configuring, running, and analyzing Delphi Surveys, CNTR initially designed the Survey to incorporate a 5-point Likert Scale. However, the NTRR Executive Steering Committee recommended an abbreviated three-response approach to reduce participant cognitive load.

The project team created an online Delphi Survey using Calibrum’s Surveylet (https://calibrum.com) application, populating it with the data elements previously identified along with their definitions and PVs, as collected after an established order of precedence.^17^ Participant engagement and project timeline were considered when setting the parameters for the number of data elements to include. By limiting the number of elements to include approximately 50 elements per environment, the team was able to maintain project feasibility, help reduce respondent burden, and improve the likelihood of sustained engagement. CNTR staff tested the surveys and made modifications to the instructions and formatting to improve clarity prior to launch. The workgroup then proceeded through three rounds of consideration in the Delphi Survey (May to August 2024). During round 1, workgroup members had the opportunity to (1) Rate whether an instrument or element should be included in the NTRR through the response options of “include,” “exclude,” or “no opinion,” (2) Rank the currently existing definition/PVs of the data element in order of preference for adoption (rank 1 being most preferred), (3) Leave comments describing their rationale regarding the inclusion of an instrument, data element, or preference of definition, and (4) Recommend additional instruments and data elements for inclusion. For each data element or measurement instrument to reach consensus for inclusion or exclusion in the NTRR, an 80% agreement threshold was set. For individual data elements, definition selection was based on the definition receiving the lowest mean ranking (ie, closest to 1). Data elements that reached consensus were hidden in subsequent rounds so that workgroup members considered only those elements that had not yet reached consensus. Email reminders were also sent periodically throughout the entire process to encourage timely completion of each round. In rounds 2 and 3, respondents saw the current consensus statistics for each item, read comments made by others, and had the option of changing their rating. If a panelist did not complete a given round, their most recent response from the previous round was retained and used in the consensus calculations for that round. This pragmatic approach helped to minimize missing data and keep the number of panelists included in calculations consistent across rounds. A total of 11 (92%) workgroup members participated in round 1, 8 (67%) in round 2, and 9 (75%) in round 3. We did not analyze the acceptable response rate prior to workgroup launch; however, routine monthly meeting attendance and frequent communication with members satisfied participation needs.

Throughout and after the Delphi process, the workgroup met monthly (May to October 2024) via teleconference to discuss outstanding items for review, including (1) Comments left by workgroup members, (2) Data elements with tied rankings on their definitions and PVs, (3) Creation of definitions/PVs for data elements that did not exist within the repositories being considered, and (4) Data elements selected for inclusion by more than one workgroup. The CDE Steering Committee and Executive Committee then reviewed and subsequently approved the results of the Delphi process.

## Results

### Outcome measurement instruments

**Figure 1:**
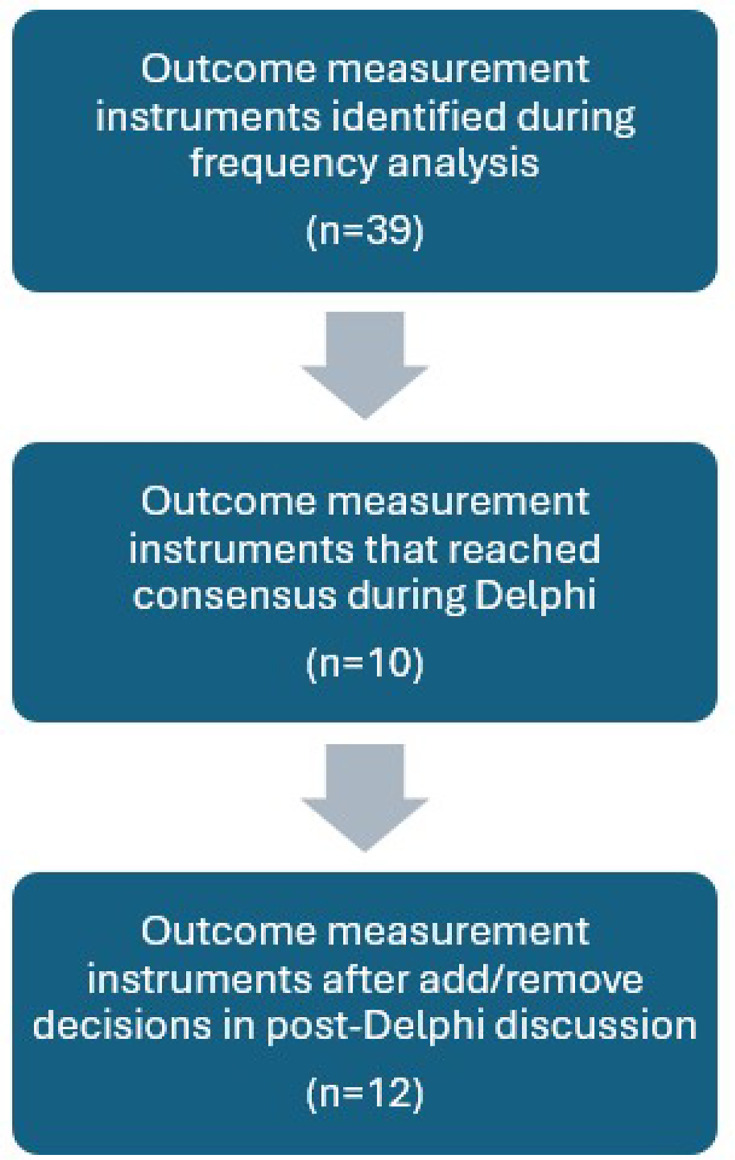
Flow diagram of outcome measurement instruments through the basic CDE selection process. CDE, common data element.

**Figure 2:**
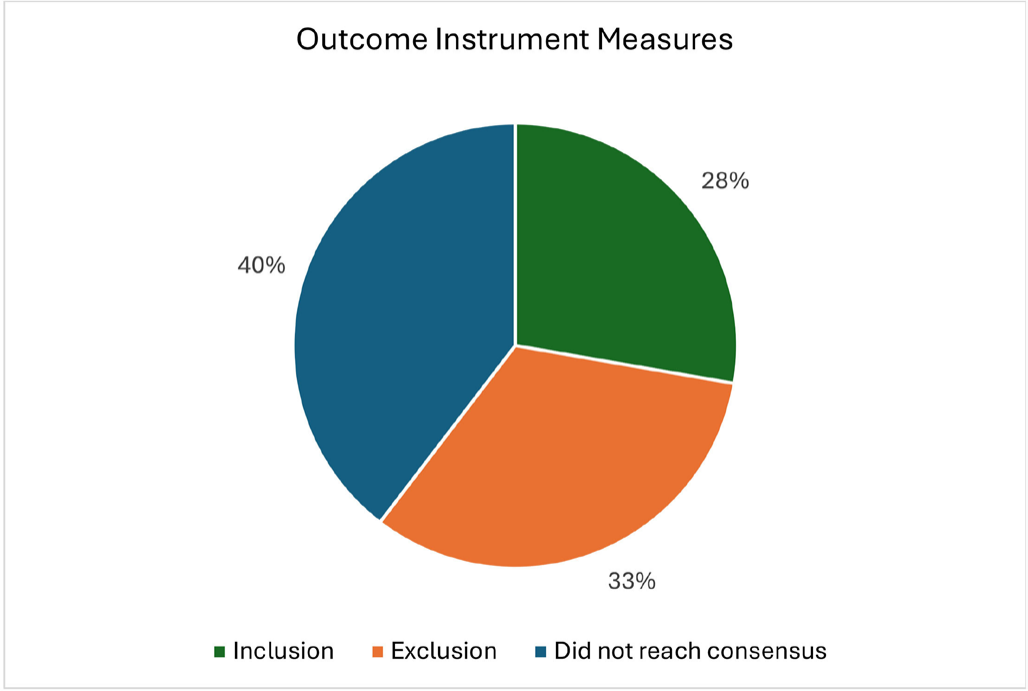
Outcome measurement instrument Delphi results.

Fifteen sources were reviewed for outcome measurement instruments, and measurement instruments that appeared two or more times in the frequency analysis were included in the Delphi Survey (online supplemental item 2).^78 11^,^14 19^ Thirty-nine measurement instruments were initially included for consideration in the Delphi Survey. After round 1, 4 more outcome measurement instruments were recommended by the workgroup members for consideration, for a total of 43 instruments. Figure 1 illustrates the flow of outcome measurement instruments through the selection process. After the three Delphi rounds and post-Delphi workgroup meetings, 12 instruments (28%) reached consensus for inclusion, 14 instruments (33%) reached consensus for exclusion, and 17 instruments (40%) did not reach consensus (figure 2). The instruments that reached consensus for inclusion are provided in box 1.

Box 1 **Outcome measurement instruments that reached consensus for inclusion in the National Trauma Research Repository (NTRR) (n=12)**

**Measurement instrument name**

Post-traumatic Stress Disorder Checklist (PCL-5)*

PROMIS Item Bank V.1.1 Pain Interference† (administered by computerized adaptive testing (CAT)) or any short form drawn from the item bank)

PROMIS Scale V.2.0 Pain Intensity 3a a †

PROMIS Item Bank V.2.0 Ability to Participate in Social Roles† (administered by CAT or any short form drawn from the item bank)

CAGE Alcohol Questionnaire‡

PROMIS Bank V.2.0 Physical Function† (administered by CAT or any short form drawn from the item bank)

Satisfaction with Life Scale (SWLS)§

PROMIS-29 Profile V.2.1†

Continuity Assessment Record and Evaluation (CARE) Functional Abilities (ie, Inpatient Rehabilitation Facility - Patient Assessment Instrument (IRF-PAI) Section GG: Functional Abilities and Goals; part of the CARE item set)¶

General Anxiety Disorder-7 (GAD-7)**

Patient Health Questionnaire-8 (PHQ-8)**

Patient Health Questionnaire-9 (PHQ-9)**

*Public domain (available from http://www.ptsd.va.gov/).†Copyright 2025 Northwestern University. Funding for Health Measures was provided by the National Institutes of Health grant U2C CA186878 (available from https://www.healthmeasures.net).‡Public domain (available from Ewing, John A. 1984. “Detecting Alcoholism: The CAGE Questionnaire.” *JAMA* 252(14):1905–1907).§Free to use for research and professional practice as long as proper credit is given to the authors (available from Diener E, Emmons RA, Larsen RJ, Griffin S. 1985. “The Satisfaction With Life Scale.” J Pers Assess 49(1):71-5).¶Copyright Centers for Medicare & Medicaid Services (CMS).**Copyright Pfizer; however, in 2010 Pfizer made this form freely available (http://www.phqscreeners.com/).PROMIS, Patient-Reported Outcomes Measurement Information System.

Together, these instruments address physical function and functional abilities, mental health (eg, anxiety, depressive symptoms), pain, participation in social life, and quality of life. There are no fees associated with the use of these measurement instruments. The majority (91%) of these instruments are patient-reported outcome measures. The instruments that reached consensus for exclusion, or did not reach consensus for either inclusion/exclusion, are provided in online supplemental item 3.

### Outcome CDEs

**Figure 3:**
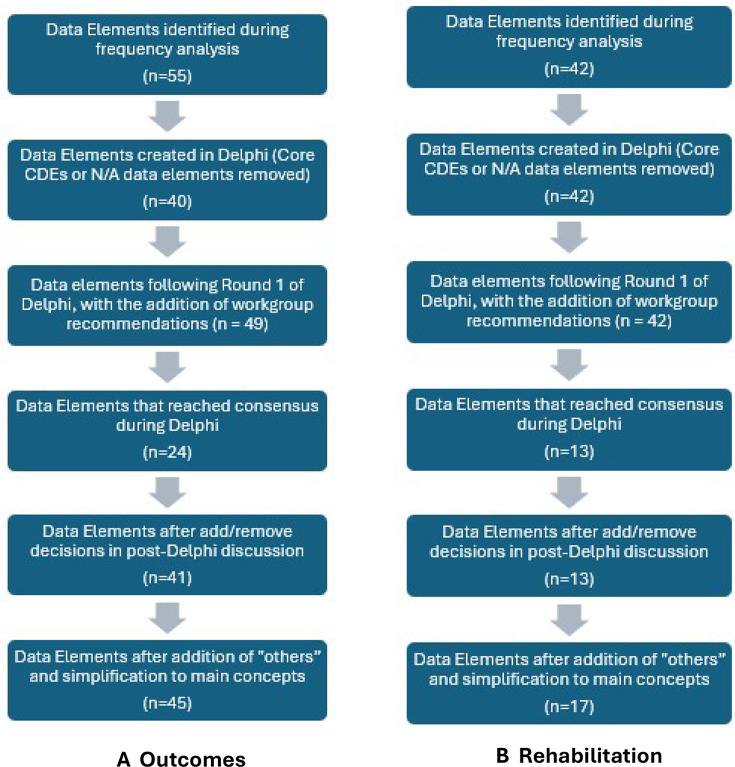
Flow diagram of individual outcome and rehabilitation data elements through the basic CDE selection process. CDE, common data element.

**Figure 4:**
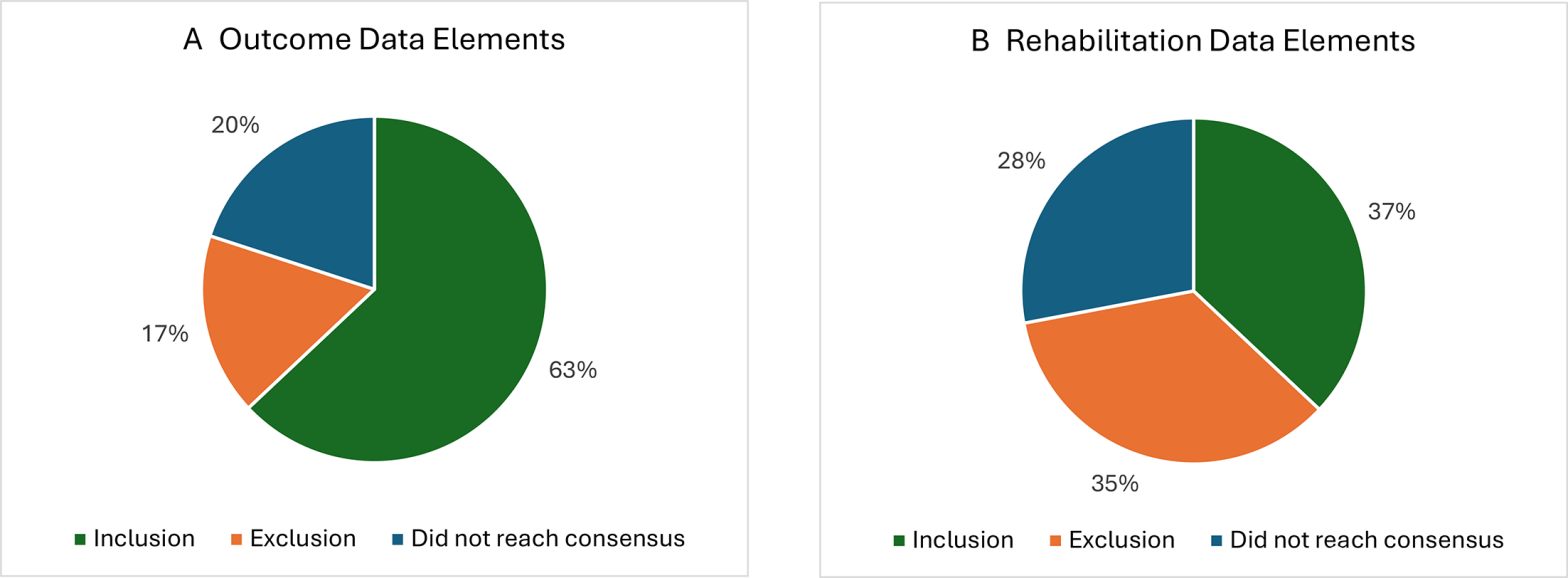
Individual outcome and rehabilitation data elements Delphi results.

Seventeen sources were reviewed for outcome data elements, and the top 55 data elements in the frequency analysis were included in the initial Delphi Survey (online supplemental item 2).^58 11^,^14 19^ Upon the removal of overlapping elements, 40 data elements remained. After round 1, nine more outcome data elements were recommended by the workgroup members for consideration. Figure 3A illustrates the flow of outcome data elements through the selection process. After the three Delphi rounds and post-Delphi workgroup meetings in which discussions surrounding goniometry measurements emerged, 45 (63%) data elements reached consensus for inclusion, 12 (17%) data elements reached consensus for exclusion, and 14 (20%) data elements did not reach consensus (figure 4A). These results directly take into account the 22 elements that target specific goniometry measurements (both active and passive), total active motion, and the individual addition of a data element that captures the “Other” branching elements—that is, data elements that capture instances in which “Other” is selected as a PV and expand on the predefined options—as these elements were not included on the initial Delphi Survey.

The outcome data elements that reached consensus for inclusion are provided in box 2. The complete listing of outcome data elements that met consensus for inclusion in the NTRR with the original definition source, data definition, input restrictions, and PVs can be found in online supplemental item 4.

Box 2 **Individual outcome and rehabilitation data elements that reached consensus for inclusion in the National Trauma Research Repository (NTRR)**

**Data element name**

**Outcomes (n=45)**

Hospital discharge date

Hospital discharge disposition

Hospital re-admissions/unplanned rehospitalization

Drug/substance use/substance abuse (general)

Employment status (postinjury/current)

Cause of death

Alcohol use/drinking habits

Return to work (general)

Postdischarge contacts with healthcare/healthcare utilization

Length of hospital re-admission/unplanned rehospitalization (ie, days)*

Education level

Employment accommodations

Physical problems/limitations

Residence type

Psychological/psychiatric illness/diagnosis†

Psychological/psychiatric illness/diagnosis specify other‡

People living with pain

Vital status

Reason for hospital re-admissions/unplanned rehospitalization

Self-rated health (general)

Re-admission indicator

Number of days to return to work

Goniometry ankle measurement (active)

Goniometry elbow measurement (active)

Goniometry hip measurement (active)

Goniometry knee measurement (active)

Goniometry neck measurement (active)

Goniometry shoulder measurement (active)

Goniometry torso measurement (active)

Goniometry wrist measurement (active)

Goniometry shoulder abduct measurement (active)

Goniometry shoulder forward flexion measurement (active)

Laterality type

Goniometry ankle measurement

Goniometry elbow measurement

Goniometry hip measurement

Goniometry knee measurement

Goniometry neck measurement

Goniometry shoulder measurement

Goniometry torso measurement

Goniometry wrist measurement

Goniometry shoulder abduct measurement

Goniometry shoulder forward flexion measurement

Total active motion (TAM)

**Rehabilitation (n=17)**

Therapy/rehabilitation frequency

Home assistance

Length of stay in rehabilitation institution /days spent in inpatient rehab

Therapy/rehabilitation admission/start date time

Therapy/rehabilitation discharge/end date time

Therapy/rehabilitation type

Therapy/rehabilitation specify other‡

Patient refusal for rehabilitation sessions

Therapy/rehabilitation activities/interventions

Therapy/rehabilitation activities/interventions specify other‡

Wheelchair or scooter use

Mobility device type specify other‡

Therapy/rehabilitation ongoing indicator

Family or caregiver involvement/participation

Telemedicine/telehealth

Other patient visit site type‡

Mechanical ventilation at discharge

*Data will be captured using the following common data elements (CDEs) approved by the Acute Workgroup: length of hospital stay in days and re-admission indicator.

†Data will be captured using the pre-existing conditions CDE approved by the Acute and Epidemiology workgroups.

‡Elements were not included in the Delphi, but were created due to “Other, specify” permissible values.

In considering the feasibility of the data elements, it was analyzed that the item for length of hospital re-admission would best be captured using the “length of hospital stay in days” and “readmission indicator” CDEs approved by the NTRR Acute Hospital Workgroup (one of four additional NTRR CDE workgroups considering elements). Furthermore, the psychological/psychiatric illness/diagnosis item could be captured using the “pre-existing conditions” CDE approved by the Acute Hospital Workgroup and Epidemiology Workgroup. Notably, the data elements for in-hospital mortality and mortality at 30 days, 90 days, and 365 days were removed from the recommended elements for inclusion, since it was analyzed that these data points could be captured using time-related CDEs (ie, “date of injury,” “date of hospital admission,” and “date of death”) that were selected during the Prehospital Workgroup and Acute Hospital Workgroup selection process.^31 32^

### Rehabilitation CDEs

Eleven sources were reviewed for rehabilitation data elements, and the 41 rehabilitation data elements that were identified in the frequency analysis were included in the Delphi Survey (online supplemental item 2).^12^,^1619 20 24 26 27 33^ Figure 3B illustrates the flow of rehabilitation data elements through the selection process. After the three Delphi rounds and post-Delphi workgroup meetings, 17 (37%) data elements reached consensus for inclusion, 16 (35%) data elements reached consensus for exclusion, and 13 (28%) data elements did not reach consensus (figure 4B). These results consider the addition of data elements that capture the “Other” branching elements.

Box 2 also outlines the rehabilitation data elements that reached consensus for inclusion. The complete listing of rehabilitation data elements that met consensus for inclusion in the NTRR with the original definition source, data definition, input restrictions, and PVs can be found in online supplemental item 4.

The outcome and rehabilitation data elements that reached consensus for exclusion, or did not reach consensus for either inclusion or exclusion in the NTRR, are also provided in online supplemental item 3.

## Discussion

The goal of this project was to identify and recommend basic CDEs for the therapeutic areas of outcomes and rehabilitation. This was achieved through the Delphi process and concurrent monthly workgroup meetings. Twelve outcome measurement instruments, 45 outcome CDEs, and 17 rehabilitation CDEs reached consensus and are now available in the NTRR data dictionary for use by trauma researchers. Widespread use of these recommended CDEs will enable the harmonization of data elements across studies, facilitating secondary analyses and reproducibility, potentially increasing knowledge that leads to improved patient outcomes. These nearly five dozen CDEs and a dozen measurement instruments are not meant to be an exhaustive listing of all elements that research in the outcomes and rehabilitation therapeutic areas should use, but should represent elements common to most studies. As the NTRR matures, additional data elements and measurement instruments may be added as needed, thus allowing the process to be iterative. As the needs of trauma researchers investigating the outcomes and rehabilitation of patients evolve, the basic CDEs for these therapeutic environments will be continually evaluated to ensure investigators’ needs are met.

There were a variety of measurement instruments and data elements that did not reach consensus for inclusion. The Patient-Reported Outcomes Measurement Information System (PROMIS) items for anxiety, depressive symptoms, fatigue, sleep disturbance, and upper extremity did not reach consensus for inclusion. The workgroup decided through discussion to limit the number of redundant measures for similar health domains. For instance, since the Patient Health Questionnaire-9 and Patient Health Questionnaire-8 were recommended for inclusion, and they both capture depression and provide the option of assessing suicidality, the full PROMIS depressive symptoms instrument was deemed unnecessary. Similarly, since the General Anxiety Disorder-7 was selected for inclusion, the full PROMIS anxiety instrument was left off the recommendations for inclusion. It should be noted, however, that the short-form PROMIS-29 Profile (V.2.1) that was selected for inclusion also includes assessment of these two health domains. In other instances, an instrument did not reach consensus for inclusion on the basis that the instrument was deemed inapplicable to most adult trauma populations. The Glasgow Outcome Scale – Extended (GOS-E) is one example. The GOS-E did not reach consensus for inclusion; although appropriate for assessing patients with traumatic brain injury (TBI), the workgroup analyzed that this measure wouldn’t be appropriate for other patient populations outside the TBI population. However, due to the NTRR’s integration with the Federal Interagency Traumatic Brain Injury Research (FITBIR) Informatics System, the GOS-E does remain accessible to researchers since it is a part of FITBIR’s data dictionary and the TBI core CDEs. Furthermore, many of the measurement instruments selected for inclusion are patient-reported outcome measures, and only one clinician-reported outcome measure was included: the Continuity Assessment Record and Evaluation (CARE) Functional Abilities. This result is likely due to the necessity of a trained healthcare professional to administer a clinician-reported outcome measure assessment;^34^ further, the ease of administration with patient-reported outcome measurement instruments helps to reduce the overall administrative burden of research and allow an instrument to be more easily and widely adopted. More specifically, the inclusion of the CARE Functional Abilities measure was likely due to the inclusion of this item on the US Centers for Medicare & Medicaid Services’ Inpatient Rehabilitation Facility - Patient Assessment Instrument, thus making it a common instrument of use. However, just because an item did not reach consensus for inclusion, or an item met consensus for exclusion, researchers are not prevented from using the instrument or element if it already exists in FITBIR as a CDE, or if the element is appropriately relevant and thus incorporated into the NTRR in the future. The items selected for inclusion through this process serve as a foundational starting point for data collection.

Of interest, goniometry for range of motion (ROM) was an item added to the Delphi Survey by recommendation of a workgroup member. Upon reaching consensus for inclusion, the workgroup reviewed the existing ways that this item had previously been operationalized. The existing goniometry variables in the NINDS CDE catalog captured active ROM for a variety of joints; however, the workgroup analyzed that it would be beneficial to capture passive ROM as well. Thus, the CDEs created for goniometry capture both passive and active ROM.

Although the recommended rehabilitation data elements emerged from the projects identified by the workgroup, examinations into what occurs throughout the inpatient and outpatient rehabilitation care environments are evolving. For instance, the Rehabilitation Treatment Specification System is a conceptual framework that begins to decipher the various treatments of rehabilitative care by identifying the organ functions, skills and habits, and representations of each intervention.^35^ This approach goes beyond the existing metrics of frequency and duration. Moreover, the ongoing Comparing Treatment Approaches to Promote Inpatient Rehabilitation Effectiveness for Traumatic Brain Injury project is exploring the fluid nature of what happens in a rehabilitation session, both at the patient and therapist levels, including factors that apply to all sessions as well as those that are unique to individual sessions.^36^ As these efforts mature, the need to incorporate additional data elements into this existing framework will become necessary. This capacity for the NTRR to evolve will ensure that this process remains iterative and that CDEs remain responsive to the needs of the trauma research community over time.

This process of selecting basic CDEs for outcomes and rehabilitation therapeutic areas using a modified Delphi approach is not without limitations. The studies and projects the NTRR Outcomes and Rehabilitation Workgroup reviewed for data elements were collected based on workgroup member knowledge and previous experience; thus, some essential studies may have been overlooked. Additionally, many of the sources included were projects conducted in the USA; therefore, the recommendations provided here may not apply to researchers and studies conducted in other countries. Although the workgroup was multidisciplinary and included members with varying research expertise, this group did not include any patient survivors, family members or other non-clinical caregiver representatives, and the resulting CDE recommendations were not reviewed by this stakeholder group. There are various benefits to survivor and caregiver partnerships when identifying outcome measures, such as the value enhancement that comes with the recommendations being relevant to these stakeholders.^37^ Although this insight is missing due to time constraints and project scope, we encourage future iterations of this work to incorporate this important perspective. Finally, an intentional effort was made to limit workgroup size to roughly a dozen participants after NIH guidance regarding effective Delphi groups. Given this and the fluctuation of workgroup participation throughout the three rounds (92% in round 1, 67% in round 2, 75% in round 3), there is potential for bias, and it is possible that other important outcome and rehabilitation metrics were missed. Despite these valid limitations, this effort reinforces the value of the Delphi method for consensus generation. The iterative survey rounds and congruent structured expert discussion allowed for refinement of the CDEs. The impact of these limitations is mitigated by the ability of the NTRR to evolve over time with the addition of other CDEs as needed to capture new study data.

As a repository that will store data collected during the course of clinical research, the NTRR, and thus the data elements and instruments recommended, are optional for adoption but not mandatory. Individual researchers are encouraged to balance technical feasibility and pragmatism when using the data elements and instruments (eg, time to complete an instrument, administration via computerized adaptive testing or short form drawn from the item bank).

Through a consensus-driven approach, the NTRR Outcomes and Rehabilitation Workgroup selected 12 outcome measurement instruments, 45 outcome CDEs, and 17 rehabilitation CDEs for inclusion in the NTRR data dictionary as basic CDEs. Next steps include dissemination of these basic CDEs for use in future trauma studies that include patient outcomes and the rehabilitation phase of care. This dissemination will involve a comprehensive outreach strategy that targets multiple audiences. Key activities will include promoting the NTRR through CNTR’s social media accounts, website (www.nattrauma.org), and e-newsletter, as well as at various scientific meetings relevant to trauma. In addition to exhibiting and distributing information at its booth, CNTR will submit abstracts for poster and podium presentations at these trauma meetings for greater exposure. CNTR member organizations will also be encouraged to promote the NTRR through their respective publications. Through quarterly webinars, targeted messages to key constituencies (via platforms such as the *Behind the Knife* podcast and the *EAST TraumaCast*), and active solicitation of studies, the awareness and implementation of these CDEs will increase within trauma research.

## Supplementary material

10.1136/tsaco-2025-002088 online supplemental file 1

10.1136/tsaco-2025-002088 online supplemental file 2

10.1136/tsaco-2025-002088 online supplemental file 3

10.1136/tsaco-2025-002088 online supplemental file 4

## Data Availability

No data are available.
