# Supplementary material for: Adopting common data elements (CDEs) for the National Trauma Research Repository (NTRR): the results of an outcome, outcome measures, and rehabilitation Delphi Survey
Source: Trauma Surg Acute Care Open. 2026 Jul 2;11(Suppl 3):e002088. doi: 10.1136/tsaco-2025-002088 (PMC13331196; doi:10.1136/tsaco-2025-002088)
Supplement: online supplemental file 2 [file tsaco-11-Suppl_3-s002.pdf]

Supplemental Item 2. Sources Reviewed and Frequency Analysis Tables

OUTCOME MEASUREMENT INSTRUMENTS SOURCES REVIEWED (N=15)

National Trauma Research Action Plan (NTRAP) AIM 2 Core Outcome Measurement Set (COMS)[1]
Consensus Conference on Trauma Patient-Reported Outcome Measures[2]
Inpatient Rehabilitation Facility - Patient Assessment Instrument (IRF-PAI)[3]
FITBIR Basic Outcome Measures[4]
Burn Model System (BMS)[5]
Burns TBI Data Dictionary (SAC MITC group)[6]
Collaborative European NeuroTrauma Effectiveness Research in Traumatic Brain Injury (CENTER-TBI)[7]
Contemporary management of right upper quadrant gunshot wounds[8]
Functional Outcomes and Recovery after Trauma Emergencies (FORTE)[9]
Major Extremity Trauma Research Consortium (METRC)[10]
The National Study on Costs and Outcomes of Trauma (NSCOT)[11]
Spinal Cord Injury Model System (SCIMS)[12]
Standard Therapy Plus Active Therapy for Severe Burns After Skin Graft Surgery (STAT Study)[13]
Transforming Research and Clinical Knowledge in Traumatic Brain Injury (TRACK-TBI)[14]
Traumatic Brain Injury Model Systems (TBIMS)[15]

Outcome Measurement Instruments Frequency Analysis
(N=39)

Outcome Measurement Instrument	Frequency	Percent
Life Impact Burn Recovery Evaluation (LIBRE) Instrument	n/a	n/a
Patient Health Questionnaire-9 (PHQ-9)	7	47%
Post-Traumatic Stress Disorder Checklist (PCL-5)	7	47%
Short Form Health Survey-12 (SF-12)	6	40%
Satisfaction With Life Scale (SWLS)	6	40%
Glasgow Outcome Scale – Extended (GOS-E)	6	40%
PROMIS (Patient-Reported Outcomes Measurement Information System) Pain Interference	4	27%
General Anxiety Disorder-7 (GAD-7)	4	27%
Alcohol Use Disorders Identification Test - Concise (AUDIT-C)	3	20%
PROMIS (Patient-Reported Outcomes Measurement Information System) Physical Function	3	20%
PROMIS (Patient-Reported Outcomes Measurement Information System) Pain Intensity	3	20%
Short Form Health Survey-36 (SF-36)	3	20%
Continuity Assessment Record and Evaluation (CARE) Functional Abilities (I.E., IRF-PAI Section Gg: Functional Abilities and Goals; Part of The “Care” Item Set)	3	20%
Rey Auditory Verbal Learning Test (RAVLT)	3	20%
Rivermead Post-Concussion Symptoms Questionnaire (RPQ)	3	20%
PROMIS (Patient-Reported Outcomes Measurement Information System) Ability to Participate in Social Roles	2	13%
NIH Toolbox General Life Satisfaction	2	13%
PROMIS (Patient-Reported Outcomes Measurement Information System) Fatigue	2	13%
PROMIS (Patient-Reported Outcomes Measurement Information System) Sleep Disturbance	2	13%
Trauma Quality of Life Instrument (T-QOL)	2	13%
Cage Alcohol Questionnaire	2	13%
Patient Health Questionnaire-8 (PHQ-8)	2	13%
PROMIS (Patient-Reported Outcomes Measurement Information System) Anxiety	2	13%
PROMIS (Patient-Reported Outcomes Measurement Information System) Depressive Symptoms	2	13%
Revised Trauma Quality of Life (RT-QOL)	2	13%
Brief Symptom Inventory - 18 Item (BSI-18)	2	13%
Brief Test of Adult Cognition by Telephone (BTACT)	2	13%
Burn Specific Health Scale-Brief (BSHS-B)	2	13%
Craig Handicap and Assessment Reporting Technique, Short Form (CHART-Sf)	2	13%
Disability Rating Scale (DRS)	2	13%
Functional Independence Measure (FIM)	2	13%
Galveston Orientation and Amnesia Test (GOAT)	2	13%
Insomnia Severity Index (ISI)	2	13%
JFK Coma Recovery Scale-Revised (CRS-R)	2	13%
Promis-29 Profile V2.1	2	13%
Quality Of Life After Brain Injury - Overall Scale (QOLIBRI-OS)	2	13%
Trail Making Test (TMT)	2	13%
Veterans Rand 12 Item Health Survey (VR-12)	2	13%
Wechsler Adult Intelligence Scale (WAIS-IV), Processing Speed Index (PSI)	2	13%

n/a – instrument was recommended by a workgroup member.

OUTCOME DATA ELEMENT SOURCES REVIEWED
(N=17)

National Trauma Data Standard (NTDS) Data Dictionary[16]
National Trauma Research Action Plan (NTRAP) AIM 2 Core Outcome Measurement Set (COMS)[1]
FITBIR Basic CDEs for Rehabilitation Studies for Moderate–Severe TBI[4]
Burn Model System (BMS)[5]
Burn Quality Improvement Program (BQIP)[17]
Burns TBI Data Dictionary (SAC MITC group)[6]
Contemporary management of right upper quadrant gunshot wounds[8]
Functional Outcomes and Recovery after Trauma Emergencies (FORTE)[9]
Lower Extremity Assessment Project (LEAP)[18]
Major Extremity Trauma Research Consortium (METRC)[10]
The National Study on Costs and Outcomes of Trauma (NSCOT)[11]
National Burn Data Standard (NBDS)[19]
Spinal Cord Injury Model System (SCIMS)[12]
Standard Therapy Plus Active Therapy for Severe Burns After Skin Graft Surgery (STAT Study)[13]
Inpatient Rehabilitation Facility - Patient Assessment Instrument (IRF-PAI)[3]
Transforming Research and Clinical Knowledge in Traumatic Brain Injury (TRACK-TBI)[14]
Traumatic Brain Injury Model Systems (TBIMS)[15]

Data Element Frequency from Outcome Data Sources
(N=55)

Data Element	Frequency	Percent
Ventilator Use / Total Days	8	47%
Education Level	8	47%
Cause Of Death (ICD, E-Code, Or Other)	8	47%
Marital Or Partner Status	8	47%
Hospital Re-Admissions / Unplanned Rehospitalization	8	47%
ICU Days (Total; Length of Stay)	7	41%
Alcohol Use / Habits / History of Alcoholism	7	41%
Length Of Stay (LOS) Hospital	7	41%
Discharge Date Hospital	6	35%
Date Of Death (+/- Time)	6	35%
Work Status (Post-Injury / Current)	6	35%
Post Discharge Contacts with Health Care / Healthcare Utilization	5	29%
Discharge Time Hospital	5	29%
Residence Type / Residential Status / Housing (General / Current)	5	29%
Residence Type / Residential Status / Housing (Post-Injury / After Rehab Discharge)	5	29%
Driving Status / Transportation Mode / Vehicles	4	24%
Self-Rated Health (General)	4	24%
Drug Use / Substance Use / Substance Abuse (General)	4	24%
Return To Work (General)	3	18%
Work Status (Pre/Post Injury Not Specified)	3	18%
Discharge Disposition Hospital	3	18%
Discharge Disposition (General)	3	18%
Discharge Destination Type	3	18%
Hospital Re-Admissions / Unplanned Rehospitalization (Length; # Days)	3	18%
Return To Work (Days to Return)	3	18%
Vital Status: Alive At Discharge	3	18%
Physical Problems / Limitations / Impairment	3	18%
Psychological Issues / Psychiatric Illness / Mental Health Diagnosis	3	18%

Data Element	Frequency	Percent
Education Years	2	12%
Mortality (In-Hospital)	2	12%
Driving: Adaptive Equipment for Driving	2	12%
Gait Speed (E.G., Determined By 4- Or 10-Meter Walk Test)	2	12%
Hospital Re-Admissions / Unplanned Rehospitalization Reason	2	12%
Hours Working for Pay/Week (Post-Injury)	2	12%
Pain	2	12%
Drug: Marijuana Use	2	12%
Employment Accommodations	2	12%
GED	2	12%
Hours Working (General)	2	12%
Reason For Not Working or Going to School	2	12%
Residence: Living With / Person Living With / People in Household (Post-Injury / After Rehab Discharge)	2	12%
Weeks/Months Working a Job (Post-Injury)	2	12%
Work Missed Because Of Illness or Injury (Frequency / Days / Months)	2	12%
Employment Stability	1	6%
Mortality (1-Year)	1	6%
Mortality (30-Days)	1	6%
Mortality (90-Days)	1	6%
Driving: Reason If Not Driving	1	6%
ED Visits Since Initial Hospital Discharge	1	6%
Gait Cadence of Steps	1	6%
Gait Length of Stride When Walking	1	6%
Hospital Re-Admissions / Unplanned Rehospitalization Total Number	1	6%
Marital Or Partner Status Change	1	6%
Return To Active Duty	1	6%
Same Work as Pre-Injury	1	6%

REHABILITATION SOURCES REVIEWED
(N=11)

FITBIR Basic CDEs for Rehabilitation Studies for Moderate–Severe TBI[4]
Burn Model System (BMS)[5]
Functional Outcomes and Recovery after Trauma Emergencies (FORTE)[9]
Inpatient Rehabilitation Facility - Patient Assessment Instrument (IRF-PAI)[3]
Shifting Rehabilitation Paradigms in Skilled Nursing Facilities[20]
Spinal Cord Injury Model System (SCIMS)[12]
Spinal Cord Injury Rehabilitation Study, United States (SCIRehab)[21]
Standard Therapy Plus Active Therapy for Severe Burns After Skin Graft Surgery (STAT Study)[13]
Transforming Research and Clinical Knowledge in Traumatic Brain Injury (TRACK-TBI)[14]
Traumatic Brain Injury Model Systems (TBIMS)[15]
Traumatic Brain Injury Practice-Based Evidence Study (TBI-PBE Study)[22]

Data Element Frequency from Rehabilitation Data Sources
(N=41)

Data Element	Frequency	Percent
Therapy/Rehabilitation Admission / Start Date Time	7	64%
Therapy/Rehabilitation Session Duration	6	55%
Therapy/Rehabilitation Discharge / End Date Time	5	45%
Co-Treatment Therapy / 2+ Different Therapy Services at The Same Time	4	36%
Therapy/Rehabilitation Type	4	36%
Length Of Stay in Rehabilitation Institution / Days Spent in Inpatient Rehab	3	27%
Rehabilitation Interruption (Short-Term)	3	27%
Days From Injury to Rehab Admit	2	18%
Factors Impacting Session	2	18%
Participation - Patient Involvement	2	18%
Patient Refusal for Rehabilitation Sessions	2	18%
Rehabilitation Interruption (Short-Term) - End Date	2	18%
Rehabilitation Interruption (Short-Term) - Start Date	2	18%
Therapy Received at Other Facility / Therapy Facility Type	2	18%
Therapy/Rehabilitation Frequency	2	18%
Therapy/Rehabilitation Ongoing Indicator	2	18%
Therapy/Rehabilitation Type - Individual or Group	2	18%
Complications During Rehabilitation	1	9%
Days From Injury to Rehab Discharge	1	9%
Days From Rehab Admit to Rehab Discharge Not Including Interruptions	1	9%
Home Assistance	1	9%

Data Element	Frequency	Percent
Level Of Assistance	1	9%
Mechanical Ventilation at Discharge	1	9%
Number Of Sessions in Past 4 Weeks	1	9%
Participation - Family or Caregiver Involvement	1	9%
Patient Directed Care - Extent	1	9%
Patient Refusal for Rehabilitation Sessions - Reason	1	9%
Short-Term Discharge Days—From Rehab Admit to System Discharge	1	9%
Short-Term Discharge Days—From System Acute Med to Rehab Admit	1	9%
Telemedicine	1	9%
Therapy Received at Burn Center	1	9%
Therapy Received Using Telehealth	1	9%
Therapy/Rehabilitation ICD 9 CM Code	1	9%
Therapy/Rehabilitation Missed	1	9%
Therapy/Rehabilitation Missed - Reason	1	9%
Therapy/Rehabilitation Missed - Time/Length	1	9%
Therapy/Rehabilitation Stopped Made Decision to Discontinue	1	9%
Therapy/Rehabilitation Stopped Reason	1	9%
Therapy/Rehabilitation Type - Did It Help?	1	9%
Wheelchair Or Scooter Use	1	9%
Wheelchair Or Scooter Use - Type Most Used	1	9%

References

1. Herrera-Escobar JP, Price MA, Reidy E, et al. Core outcome measures for research in traumatic injury survivors: The National Trauma Research Action Plan modified Delphi consensus study. *Journal of Trauma and Acute Care Surgery*. 2022;92(5):916-923. doi:10.1097/ta.0000000000003546
2. Sakran JV, Ezzeddine H, Schwab CW, et al. Proceedings from the Consensus Conference on Trauma Patient-Reported Outcome Measures. *Journal of the American College of Surgeons*. 2020;230(5):819-835. doi:10.1016/j.jamcollsurg.2020.01.032
3. INPATIENT REHABILITATION FACILITY - PATIENT ASSESSMENT INSTRUMENT
4. Hicks R, Giacino J, Harrison-Felix C, Manley G, Valadka A, Wilde EA. Progress in developing common data elements for traumatic brain injury research: version two--the end of the beginning. *Journal of neurotrauma*. Nov 15 2013;30(22):1852-61. doi:10.1089/neu.2013.2938
5. Burn Model System National Data and Statistical Center. Data from: Burn Injury Model System National Database. 2024.
6. American Burn Association. Burns TBI Data Dictionary (Microsoft Excel; Unpublished) 2021.
7. Maas AIR, Menon DK, Steyerberg EW, et al. Collaborative European NeuroTrauma Effectiveness Research in Traumatic Brain Injury (CENTER-TBI): A Prospective Longitudinal Observational Study. *Neurosurgery*. 2015;76(1)
8. Vella MA, Dumas RP. Contemporary Management of Right Upper Quadrant Gunshot Wounds EAST MULTICENTER STUDY DATA COLLECTION TOOL/DATA DICTIONARY. https://www.east.org/research/east-multicenter-trials/active-multicenter-trials-not-enrolling-centers: The Eastern Association for the Surgery of Trauma; University of Rochester; 2020.
9. Functional Outcomes and Recovery after Trauma Emergencies (FORTE). FORTE Data Dictionary and Questionnaire 3.0. Unpublished.
10. Castillo RC, Mackenzie EJ, Bosse MJ. Measurement of functional outcomes in the Major Extremity Trauma Research Consortium (METRC). *The Journal of the American Academy of Orthopaedic Surgeons*. 2012;20 Suppl 1:S59-63. doi:10.5435/jaaos-20-08-s59
11. Mackenzie EJ, Rivara FP, Jurkovich GJ, et al. The National Study on Costs and Outcomes of Trauma. *J Trauma*. Dec 2007;63(6 Suppl):S54-67; discussion S81-6. doi:10.1097/TA.0b013e31815acb09
12. National Spinal Cord Injury Statistical Center. Data from: Spinal Cord Injury Model System Database 2024.
13. American Burn Association. STAT: Standard Therapy Plus Active Therapy to Improve Mobility, Long-Term Activity, and Quality of Life for Severely Burn Injured Patients After Skin Graft Surgery. ClinicalTrials.gov ID: NCT043681172024.
14. Transforming Research and Clinical Knowledge in Traumatic Brain Injury (TRACK-TBI) Case Report Forms. 2024.
15. Center TBIMSNDaS. Data from: Traumatic Brain Injury Model Systems. 2024.
16. American College of Surgeons Trauma Programs. National Trauma Data Standard (NTDS) Data Dictionary. 2024.
17. American Burn Association. Burn Quality Improvement Program Outcome and Process Measures of Care. 2016.
18. MacKenzie EJ, Bosse MJ, Pollak AN, et al. Long-term persistence of disability following severe lower-limb trauma. Results of a seven-year follow-up. *The Journal of bone and joint surgery American volume*. Aug 2005;87(8):1801-9. doi:10.2106/jbjs.E.00032
19. American Burn Association, National Burn Repository. American Burn Association National Burn Data Standard: Data Dictionary. 2015.

20. U.S. Department of Veterans Affairs, Office of Research and Development. Shifting Rehabilitation Paradigms in Skilled Nursing Facilities. ClinicalTrials.gov ID: NCT029271712020.
21. Whiteneck GG. Data from: Spinal Cord Injury Rehabilitation Study, United States, 2007-2010. 2018. doi:10.3886/ICPSR36724.v1
22. Horn SD, Corrigan JD, Bogner J, et al. Traumatic Brain Injury-Practice Based Evidence Study: Design and Patients, Centers, Treatments, and Outcomes. *Arch Phys Med Rehabil*. Aug 2015;96(8 Suppl):S178-96.e15. doi:10.1016/j.apmr.2014.09.042
