# Supplementary material for: Adopting common data elements (CDEs) for the National Trauma Research Repository (NTRR): the results of an outcome, outcome measures, and rehabilitation Delphi Survey
Source: Trauma Surg Acute Care Open. 2026 Jul 2;11(Suppl 3):e002088. doi: 10.1136/tsaco-2025-002088 (PMC13331196; doi:10.1136/tsaco-2025-002088)
Supplement: online supplemental file 3 [file tsaco-11-Suppl_3-s003.pdf]

Supplemental Item 3. Items that Reached Consensus for Exclusion or Did Not Reach Consensus

Table 1. Outcome Measurement Instruments That Reached Consensus for Exclusion or Did Not Reach Consensus in the NTRR

**Measurement Instrument Name**
**Consensus Reached for Exclusion (n=14)**
Insomnia Severity Index (ISI)
Quality of Life After Brain Injury - Overall Scale (QoLIBRI-OS)
Rivermead Post-Concussion Symptoms Questionnaire (RPQ)
Trail Making Test (TMT)
Wechsler Adult Intelligence Scale (WAIS-IV), Processing Speed Index (PSI)
Rey Auditory Verbal Learning Test (RAVLT)
Galveston Orientation and Amnesia Test (GOAT)
JFK Coma Recovery Scale-Revised (CRS-R)
Craig Handicap and Assessment Reporting Technique, Short Form (CHART-SF)
Trauma Quality of Life Instrument (T-QoL)
Short Form Health Survey-36 (SF-36)
Functional Gait Assessment (FGA)
Tinetti Performance-Oriented Mobility Assessment (POMA)
Timed Up and Go (TUG)
**Instruments that Did Not Reach Consensus (n=17)**
Alcohol Use Disorders Identification Test - Concise (AUDIT-C)
PROMIS Anxiety
PROMIS Depressive Symptoms
PROMIS Fatigue
PROMIS Sleep Disturbance
Glasgow Outcome Scale Extended (GOS-E)
Veterans RAND 12 Item Health Survey (VR-12)
Brief Test of Adult Cognition by Telephone (BTACT)
Burn Specific Health Scale-Brief (BSHS-B)
Short Form Health Survey-12 (SF-12)
NIH Toolbox General Life Satisfaction
PROMIS Upper Extremity
Revised Trauma Quality of Life (RT-QoL)
Brief Symptom Inventory - 18 Item (BSI-18)
Functional Independence Measure (FIM)
Disability Rating Scale (DRS)
Life Impact Burn Recovery Evaluation (LIBRE) Instrument

PROMIS: “Patient-Reported Outcomes Measurement Information System”

Table 2. Outcome and Rehabilitation Data Elements That Reached Consensus for Exclusion or Did Not Reach Consensus in the NTRR

Outcomes	Rehabilitation
**Data Element Name**	Data Element Name
Consensus Reached for Exclusion (n=12)	***Consensus Reached for Exclusion (n=16)***
Transfer/Process (As Relates to Outcome)	Rehabilitation Interruption (Short-Term)
Weeks/Months Working a Job (Post-Injury)	Rehabilitation Interruption (Short-Term) - Start Date
GED	Rehabilitation Interruption (Short-Term) - End Date
Hours Working (Post-Injury)	Therapy/Rehabilitation Type - Individual or Group
Hours Working (General)	Therapy Co-Treatment
Cadence Of Steps (Gait)	Time/Length of Missed Therapy/Rehabilitation
Length Of Stride When Walking (Gait)	If Therapy/Rehabilitation Stopped, Who Made Decision to Discontinue
Daily Calories Used	Factors Impacting Session
Marijuana Use	Wheelchair Or Scooter Use - Type Most Used
Ambulated Distance (In 6-Minute Walk Test)	Short-Term Discharge Days -- From System Acute Med to Rehab Admit
Ambulation; Walk 150 Feet	Reason Therapy/Rehabilitation Was Missed
Daily Distance Walked	Therapy/Rehabilitation Type - Did It Help?
Data elements that Did Not Reach Consensus (n=14)	Rehabilitation Services at A Veterans Affairs Facility
Hospital Discharge Time	Extent To Which Patient Directed Care
Driving Status / Transportation Mode	Reason That Therapy/Rehabilitation Was Stopped
Education Years	Short-Term Discharge Days -- From Rehab Admit to System Discharge
Ambulation; One Flight of Stairs	***Data elements that Did Not Reach Consensus (n=13)***
Ambulation; Walk One Street Block	Level Of Assistance During Therapy
Reason For Not Working or Going to School	Patient Involvement/Participation
Amount Of Work Missed Because Of Illness or Injury	Therapy/Rehabilitation Missed
Daily Steps Taken; Total	Therapy Received at Facility Type
Use Of Adaptive Equipment for Driving	Therapy/Rehabilitation Session Duration
Gait Speed	Complications During Rehabilitation
In-Hospital Mortality▼	Therapy/Rehabilitation ICD 10 CM Code
1-Year Mortality▼	Therapy Received at Burn Center
30-Day Mortality▼	Number Of Sessions in Past 4 Weeks
90-Day Mortality▼	Reason That a Patient Refused Rehabilitation Sessions
	Days From Injury to Rehab Admit †
	Days From Injury to Rehab Discharge †
	Days From Rehab Admit to Rehab Discharge Not Including Interruptions †

▼Data Elements were removed during workgroup discussion since the data can be captured using time-related CDEs (Date of Injury, Date of Hospital Admission, Date of Death)

† Data Elements were removed during workgroup discussion since the data can be captured using date/time CDEs (Date of Injury, Therapy/Rehabilitation Admission/Start Date Time, Therapy/Rehabilitation Admission/End Date Time
