# Supplementary material for: Adopting common data elements (CDEs) for the National Trauma Research Repository (NTRR): the results of an outcome, outcome measures, and rehabilitation Delphi Survey
Source: Trauma Surg Acute Care Open. 2026 Jul 2;11(Suppl 3):e002088. doi: 10.1136/tsaco-2025-002088 (PMC13331196; doi:10.1136/tsaco-2025-002088)
Supplement: online supplemental file 4 [file tsaco-11-Suppl_3-s004.pdf]

Supplemental Item 4.

Outcome and Rehabilitation Data Elements That Met Consensus for Inclusion in the NTRR With Original Definition Source, Data Definition, Input Restrictions and Permissible Values

Table 1. Outcome data elements that met consensus for inclusion in the NTRR with original definition source, data definition, input restrictions and permissible values

DATA ELEMENT	DEFINITION SOURCE	VARIABLE TITLE	DATA DEFINITION	INPUT RESTRICTIONS & PERMISSIBLE VALUES
Hospital discharge date	FITBIR	Hospital discharge date and time	Date (and time, if applicable and known) the participant/subject was discharged from the hospital	Date or Date & Time ISO 8601 (Free-Form Entry)
Hospital discharge disposition	NTDS	Hospital Discharge Disposition	The disposition of the patient when discharged from the hospital	Discharged/Transferred to a short-term general hospital for inpatient care | Discharged/Transferred to an Intermediate Care Facility (ICF) | Discharged/Transferred to home under care of organized home health service | Left against medical advice or discontinued care | Deceased/Expired | Discharged to home or self-care (routine discharge) | Discharged/Transferred to Skilled Nursing Facility (SNF) | Discharged/Transferred to hospice care | Discharged/Transferred to court/law enforcement. | Discharged/Transferred to inpatient rehab or designated unit | Discharged/Transferred to Long Term Care Hospital (LTCH) | Discharged/Transferred to a psychiatric hospital or psychiatric distinct part unit of a hospital | Discharged/Transferred to another type of institution not definedelsewhere
Cause of death	FITBIR	Death cause ICD-10-CM* code	ICD-10-CM* code that describes the cause of participant/subject's death	Alphanumeric (Free-Form Entry)
Vital status	NTDS	Vital status type	The type of vital status held by the patient on discharge	Alive | Dead
Hospital readmissions / unplanned rehospitalization	NINDS	Hospitalization past year count	Count of all hospitalizations the participant/subject had within the past year	Alphanumeric (Free-Form Entry)

DATA ELEMENT	DEFINITION SOURCE	VARIABLE TITLE	DATA DEFINITION	INPUT RESTRICTIONS & PERMISSIBLE VALUES
Reason for hospital readmissions / unplanned rehospitalization	FITBIR	Hospitalization reason	Reason why the participant/subject was hospitalized, excluding all surgeries	Pneumonia or Respiratory | Failure to Thrive | Dehydration | Other, specify | Trauma | Infection other than pneumonia | Fracture | Seizures | Cardiomyopathy/Arrhythmia
Hospitalization other text	FITBIR	Hospitalization other text	The free-text field related to 'Hospitalization reason' specifying other text. Reason why the participant/subject was hospitalized, excluding all surgeries.	Alphanumeric (Free-Form Entry)
Post discharge contacts with health care / healthcare utilization	caDSR	Patient Other Healthcare Service Visit Count	The count or number related to the patients visits to other health care services.	Numeric (Free-Form Entry)
Self-rated health (general)	NIH/NLM	Self-Reported Health	Self-reported health, also described as self-rated health or self-assessed health, is an individual appraisal of health status based upon individual perspectives	Excellent | Very Good | Good | Fair | Poor
Psychological/psychiatric illness/diagnosis	FITBIR	Pre-existing conditions	A condition/disease(occurring prior to injury) reported by the participant/subject or documented in the medical record as part of medical history	Advance Directive Limiting Care | Alcohol Use Disorder | Anticoagulant Therapy | Attention Deficit Disorder/Attention Deficit Hyperactivity Disorder (ADD/ADHD) | Bipolar I/II Disorder | Bleeding Disorder | Cerebral Vascular Accident (CVA) | Chronic Obstructive Pulmonary Disease (COPD) | Chronic Renal Failure | Cirrhosis | Congenital Anomalies | Congestive Heart Failure (CHF) | Current Smoker | Currently Receiving Chemotherapy for Cancer | Dementia | Diabetes Mellitus | Disseminated Cancer | Functionality Dependent Health Status | Hypertension | Major Depressive Disorder | Myocardial Infarction (MI) | Other Mental/personality Disorders | Peripheral Arterial Disease (PAD) | Post-traumatic Stress Disorder | Pregnancy | Prematurity | Schizoaffective Disorder | Schizophrenia | Steroid Use | Substance Use Disorder | Other, specify | Post-traumatic stress disorder | Anxiety | Sleep disorder
Psychological/psychiatric illness/diagnosis other text	FITBIR	Medical history psychiatric category other text	Medical history psychiatric category other text	Alphanumeric (Free-Form Entry)

DATA ELEMENT	DEFINITION SOURCE	VARIABLE TITLE	DATA DEFINITION	INPUT RESTRICTIONS & PERMISSIBLE VALUES
Physical problems / limitations	BurnModelSystem	Physical problems at follow-up	Do you currently have any physical problems, such as mobility impairment (difficulty moving your arms, legs, or body)?	Yes | No |Declined to answer/Refused |Missing/Unknown
Pain	FITBIR	Pain day past week number	Number of days with pain over the last seven days including today.	None | One day | Two days | Three days | Four days | Five days | Six days | Seven days | Unknown
Alcohol use / drinking habits	FITBIR	Alcohol consume six or more drinks frequency	The rate of occurrence for when the participant/subject consumes six or more drinks containing alcohol on one occasion. It is noted that the meaning of "drinks consumed" differs from one nation and culture to another, so the most common alcoholic beverages likely to be consumed will be defined as well as quantity of each that constitutes a drink (approximately 10 grams of pure ethanol). For example, one bottle of beer (330 ml at 5% ethanol), a glass of wine (140 ml at 12% ethanol), and a shot of spirits (40 ml at 40% ethanol) represent a standard drink of about 13 g of ethanol.	Daily or almost daily | Less than monthly | Monthly | Never | Unknown | Weekly

DATA ELEMENT	DEFINITION SOURCE	VARIABLE TITLE	DATA DEFINITION	INPUT RESTRICTIONS & PERMISSIBLE VALUES
Drug /substance use/substance abuse (general)	FITBIR	Drug or substance current illicit use indicator	Indicator of participant's/subject's use of illegal drugs, prescription or over-the-counter drugs in the past year for purposes other than those for which they are meant to be used, or in large amounts	No | Unknown | Yes
Employment status(post-injury/ current)	FITBIR	Employment status	Status of participant/subject's current employment	Disabled, permanently or temporarily | Keeping house | Looking for work, unemployed | Only temporarily laid off | Other, specify | Retired | Sick leave or maternity leave | Student | Unknown | Working Now
Employment other text	FITBIR	Employment other text	The free-text field related to 'Employment status' specifying other text. Status of participant/subject's current employment.	Alphanumeric (Free-Form Entry)
Return to work (general)	FITBIR	Return work school status	Status of participant's/subject's return to work and/or school	Did not return to work or school | Different work or school | N/A | Only in sheltered environment | Returned to previous level | Same work or school, reduced level | Unknown
Employment accommodations	NINDS	Work accommodation indicator	Indicator related to whether patient received some type of work accommodation	No | Yes

DATA ELEMENT	DEFINITION SOURCE	VARIABLE TITLE	DATA DEFINITION	INPUT RESTRICTIONS & PERMISSIBLE VALUES
Education level	FITBIR	Education level USA type	Highest grade or level of school participant/subject has completed or the highest degree received	1st Grade | 2nd Grade | 3rd Grade | 4th Grade | 5th Grade | 6th Grade | 7th Grade | 8th Grade | 9th Grade | 10th Grade | 11th Grade | 12th Grade, no diploma | High school graduate | GED or equivalent | Some college, no degree | Associate degree: academic program | Associate degree: occupational/technical/vocational program less | Bachelor's degree (e.g., BA, AB, BS, BBA) | Master's degree (e.g., MA, MS, MEng, MEd, MBA) | Doctoral degree (e.g., PhD, EdD) | Professional school degree (e.g., MD, DDS, DVM, JD) | Never attended/Kindergarten only | Other, specify | Unknown
Residence type (post-injury/after rehab discharge)	FITBIR	Residence type	Type of residence where the participant/subject is currently living	Home | Hospital | N/A - patient died | Nursing home | Other, specify | Rehabilitation center | Unknown
People living with	FITBIR	Living with person relationship type	Type(s) of relationship(s) that obtain between the participant/subject and all people with whom she or he currently lives, cohabits, or stays	Adopted son or daughter | Biological son or daughter | Brother or sister | Father or mother | Foster child | Grandchild | Grandparent | Housemate or roommate | Husband or wife | Military unit member | N/A - Alone | N/A - Homeless | Other nonrelative | Other patient/ resident in care facility | Other relative | Parent-in-law | Personal care attendant | Roomer or boarder | Son-in-law or daughter-in-law | Stepfather or stepmother | Stepson or stepdaughter | Unknown | Unmarried partner

DATA ELEMENT	DEFINITION SOURCE	VARIABLE TITLE	DATA DEFINITION	INPUT RESTRICTIONS & PERMISSIBLE VALUES
Readmission Indicator	N/A	Readmission Indicator	Indication of if the hospital stay was related to a readmission.	No | Yes | Unknown | N/A
Number of days to return to work	FITBIR	Return to work date	Date the participant returned to work following their injury	Date or Date & Time ISO 8601 (Free-Form Entry)
Goniometry ankle (active)	NTRR Workgroup	Goniometry ankle measurement (active)	Measurement in degrees of the active range of motion for ankle	Numeric (Free-Form Entry)
Goniometry elbow (active)	NTRR Workgroup	Goniometry elbow measurement (active)	Measurement in degrees of the active range of motion for elbow	Numeric (Free-Form Entry)
Goniometry hip (active)	NTRR Workgroup	Goniometry hip measurement (active)	Measurement in degrees of the active range of motion for hip	Numeric (Free-Form Entry)
Goniometry knee (active)	NTRR Workgroup	Goniometry knee measurement (active)	Measurement in degrees of the active range of motion for knee	Numeric (Free-Form Entry)
Goniometry neck (active)	NTRR Workgroup	Goniometry neck measurement (active)	Measurement in degrees of the active range of motion forneck	Numeric (Free-Form Entry)
Goniometry shoulder (active)	NTRR Workgroup	Goniometry shoulder measurement (active)	Measurement in degrees of the active range of motion for shoulder abduction	Numeric (Free-Form Entry)
Goniometry torso (active)	NTRR Workgroup	Goniometry torso measurement (active)	Measurement in degrees of the active range of motion for torso	Numeric (Free-Form Entry)
Goniometry wrist (active)	NTRR Workgroup	Goniometry wrist measurement (active)	Measurement in degrees of the active range of motion for wrist	Numeric (Free-Form Entry)
Goniometry shoulder abduct (active)	NTRR Workgroup	Goniometry shoulder abduct measurement (active)	Measurement in degrees of the active range of motion for shoulder abduction	Numeric (Free-Form Entry)
Goniometry shoulder forward flexion (active)	NTRR Workgroup	Goniometry shoulder forward flexion measurement (active)	Measurement in degrees of the active range of motion for shoulder forward flexion	Numeric (Free-Form Entry)
Laterality type	NINDS	Laterality type	Laterality type relative to the anatomic site of the body examined or affected	Left | Right

DATA ELEMENT	DEFINITION SOURCE	VARIABLE TITLE	DATA DEFINITION	INPUT RESTRICTIONS & PERMISSIBLE VALUES
Goniometry ankle (passive)	NINDS	Goniometry ankle measurement	Measurement in degrees of the passive range of motion for ankle	Numeric (Free-Form Entry)
Goniometry elbow (passive)	NINDS	Goniometry elbow measurement	Measurement in degrees of the passive range of motion for elbow	Numeric (Free-Form Entry)
Goniometry hip (passive)	NINDS	Goniometry hip measurement	Measurement in degrees of the passive range of motion forhip	Numeric (Free-Form Entry)
Goniometry knee (passive)	NINDS	Goniometry knee measurement	Measurement in degrees of the passive range of motion for knee	Numeric (Free-Form Entry)
Goniometry neck (passive)	NINDS	Goniometry neck measurement	Measurement in degrees of the passive range of motion for neck	Numeric (Free-Form Entry)
Goniometry shoulder (passive)	NINDS	Goniometry shoulder measurement	Measurement in degrees of the passive range of motion for shoulder abduction	Numeric (Free-Form Entry)
Goniometry torso (passive)	NINDS	Goniometry torso measurement	Measurement in degrees of the passive range of motion for torso	Numeric (Free-Form Entry)
Goniometry wrist (passive)	NINDS	Goniometry wrist measurement	Measurement in degrees of the passive range of motion for wrist	Numeric (Free-Form Entry)
Goniometry shoulder abduct (passive)	NINDS	Goniometry shoulder abduct measurement	Measurement in degrees of the passive range of motion for shoulder abduction	Numeric (Free-Form Entry)
Goniometry shoulder forward flexion (passive)	NINDS	Goniometry shoulder forward flexion measurement	Measurement in degrees of the passive range of motion for shoulder forward flexion	Numeric (Free-Form Entry)
Total Active Motion (TAM)	NTRR Workgroup	Total Active Motion (TAM)	Measurements of the distal interphalangeal (DIP), proximal interphalangeal (PIP), and metatarsophalangeal (MCP) joints maximally flexed. The total amount of flexion is added, and one sum is used as total active flexion of that digit. If the joints lack full extension, the deficit is subtracted from the total active flexion yielding the total active motion or TAM.	Numeric (Free-Form Entry)

[Page Intentionally Left Blank]

Table 2. Rehabilitation data elements that met consensus for inclusion in the NTRR with original definition source, data definition, input restrictions and permissible values

DATA ELEMENT	DEFINITION SOURCE	VARIABLE TITLE	DATA DEFINITION	INPUT RESTRICTIONS & PERMISSIBLE VALUES
Therapy/rehabilitation type	FITBIR	Therapy or rehabilitation type	Type of therapy or rehabilitation services received by the participant/subject	Adaptive physical education | Behavior support plan | Crisis management plan | Dietary | Occupational therapy | Physical therapy | Psychological | Recreational | Sensory integration therapy | Social skills training | Speech therapy | Vocational | Other, specify
Therapy or rehabilitation other text	FITBIR	Therapy or rehabilitation other text	The free-text field related to 'Therapy or rehabilitation type' specifying other text. Type of therapy or rehabilitation services received by the participant/subject.	Alphanumeric (Free-Form Entry)

DATA ELEMENT	DEFINITION SOURCE	VARIABLE TITLE	DATA DEFINITION	INPUT RESTRICTIONS & PERMISSIBLE VALUES
Therapy/rehabilitation activities/interventions	NINDS	Activity type	The types of activities related to rehabilitation services	Aquatic exercises | Assessment/evaluation | Assistive technology | Balance | Bathing | Bed mobility | Bladder management | Bowel management | Cognitive-communication interventions | Communication interventions | Community/in- house services |Community re-integration outing | Complementary approaches | Complications (education)|Discharge planning | Discharge services | Dressing- lower body | Dressing- upper body | Education not covered by other activities | Endurance | Equipment evaluation | Equipment evaluation/provision/education | Financial planning | Gait | Grooming | Home management skills | Leisure education and counseling | Leisure skills in center | Leisure skills in outing | Medication (education)| Modalities | Motor speech and/or voice disorder interventions | Musculoskeletal treatments/modalities | Other, specify |Other therapeutic activities | Pain education | Peer/advocacy groups | Pre-gait | Psychoeducation intervention | Psychosocial support | Psychotherapeutic intervention | Range of motion/stretching | Safety (education)| Self-feeding | Skin management | Social activity | Splint/cast fabrication | Supportive counseling | Swallowing interventions/feeding trials | Team and patient/family conferences | Team process (interdisciplinary team interactions/planning)| Toileting for clothing management and hygiene | Tracheostomy tube and/or ventilator support interventions | Transfers | Upright activities | Wheelchair mobility- manual | Wheelchair mobility- power | Woundcare
Therapy/rehabilitation activities/interventions other text	NINDS	Activity other text	The free text field related to 'Activity type' specifying other text.	Alphanumeric (Free-Form Entry)

DATA ELEMENT	DEFINITION SOURCE	VARIABLE TITLE	DATA DEFINITION	INPUT RESTRICTIONS & PERMISSIBLE VALUES
Therapy/rehabilitation admission/start date time	FITBIR	Therapy rehabilitation start date time	Date (and time, if applicable and known) on which the therapy or rehabilitation started	Date or Date & Time ISO 8601 (Free-Form Entry)
Therapy/rehabilitation discharge/end date time	FITBIR	Therapy rehabilitation end date time	Date (and time, if applicable and known) on which the therapy or rehabilitation ended	Date or Date & Time ISO 8601 (Free-Form Entry)
Therapy/rehabilitation frequency	FITBIR	Therapy rehabilitation frequency	Frequency the participant/subject received the therapy or rehabilitation (days/week)	0 | 1| 2| 3| 4| 5| 6| 7
Length of stay in rehabilitation institution / days spent in inpatient rehab	caDSR	Person Rehabilitation Facility Length Day Count	Number of days spent in a rehabilitation facility	Numeric (Free-Form Entry)
Therapy/rehabilitation ongoing indicator	FITBIR	Therapy rehabilitation ongoing indicator	Indicator of whether the therapy or rehabilitation is ongoing	No | Unknown | Yes
Telemedicine/telehealth	caDSR	Patient Visit Site Type	Information related to the site of visit by a patient to a medical professional.	Email to provider | Emergency Department | Hospital Inpatient Department | Hospital Outpatient Department | Intensive Care Unit (ICU) Hospital Inpatient Department | Other | Phone call to provider | Provider's Office | Telemedicine
Other patient visit site type	N/A	Patient Visit Site Type Other Text	The free-text field related to 'Patient Visit Site Type' specifying other text.	Alphanumeric (Free-Form Entry)
Family or caregiver involvement/participation	NINDS	Therapy participant other text	Text describing the any other people participating in the therapy session.	Alphanumeric (Free-Form Entry)

DATA ELEMENT	DEFINITION SOURCE	VARIABLE TITLE	DATA DEFINITION	INPUT RESTRICTIONS & PERMISSIBLE VALUES
Patient refusal for rehabilitation sessions	TRACK-TBI (FITBIR UDE)	No rehab follow up not interested reason type	The reasons the subject is not interested in follow-up care when they did not receive any rehabilitation treatment to address problems related to their brain injury following their ED/visit/discharge from the hospital.	Because I did not think I needed it | Because I believe I can manage the problems caused by my injury on my own | Because I was dissatisfied with the treatment I received at the ED/hospital
Home assistance	FITBIR	Paid assistance per day hours number	How many hours in a typical 24-hour day do you have someone with you [paid assistance] to provide physical assistance for personal care activities such as eating, bathing, dressing, toileting and mobility?	Numeric (Free-Form Entry)
Wheelchair or scooter use	NINDS	Mobility device type	Type for all mobility devices currently used by the participant/subject	Manual wheelchair | Other, specify | Power assist wheelchair | Power wheelchair | Quad cane | Scooter | Standing Dani | Straight cane | Stroller | Walker
Mobility device type other text	NINDS	Mobility device other text	The free-text field related to 'Mobility device type' specifying other text. Type for all mobility devices currently used by the participant/subject.	Alphanumeric (Free-Form Entry)
Mechanical ventilation at discharge	FITBIR	Ventilator assistance utilization indicator	Indicator for the utilization of ventilator assistance on discharge	No | Yes, 24 hours per day at discharge | Yes, less than 24 hours per day at discharge | Yes, unknown
